# Prognostic performance of blood neurofilament light chain for functional outcomes in patients with ischemic stroke: a systematic review and meta-analysis

**DOI:** 10.3389/fneur.2026.1895703

**Published:** 2026-10-12

**Authors:** Tong Tong He, Bo Wang, Jing Wang, Meng Li

**Affiliations:** Neurology Department, Cangzhou Central Hospital of Hebei Medical University, Cangzhou City, China

**Keywords:** functional outcome, ischemic stroke, meta-analysis, neurofilament light chain, systematic review

## Abstract

**Introduction:**

This meta-analysis aimed to assess the association between blood neurofilament light chain (NfL) and functional outcomes in patients with ischemic stroke.

**Methods:**

PubMed, Web of Science, Embase, Cochrane Library, and the China National Knowledge Infrastructure database were searched from inception through March 15, 2026. Odds ratios (ORs) and 95% confidence intervals (CIs) were pooled to quantify the association between blood NfL and functional outcomes in patients with ischemic stroke. We pooled the area under the curve (AUC), sensitivity, and specificity; conducted meta-regression and sensitivity analyses; and assessed publication bias.

**Results:**

A total of 18 studies involving 3,448 patients were included in this meta-analysis. The pooled results demonstrated that blood NfL was associated with poor functional outcomes after ischemic stroke (OR = 1.78, 95% CI: 1.41–2.24, *p* < 0.001, *I*^2^ = 87.5%, *p* < 0.01). The pooled AUC for blood NfL in predicting functional outcomes in patients with ischemic stroke was 0.79 (95% CI: 0.75–0.82), with 71% (95% CI: 62–79%) sensitivity and 74% (95% CI: 67–80%) specificity. Subgroup analyses and meta-regression revealed that participant age, sample size, study design, blood sample type, assay method, proportion of poor functional outcomes, blood sampling time, reperfusion therapy rate, National Institutes of Health Stroke Scale score, definition of poor functional outcome, and the proportion of large artery atherosclerosis were not identified as significant sources of heterogeneity. Sensitivity analysis indicated that the pooled estimate was robust. Begg’s test also showed no evidence of publication bias (*p* = 0.753), although Egger’s test suggested the presence of publication bias (*p* < 0.001). The trim-and-fill method showed that the overall effect of this meta-analysis was stable.

**Conclusion:**

This meta-analysis showed that elevated blood NfL levels may have prognostic value for functional outcomes in patients with ischemic stroke. However, given the substantial between-study heterogeneity and the presence of publication bias, these findings should be interpreted with caution.

**Systematic review registration:**

https://www.crd.york.ac.uk/PROSPERO/view/CRD420261297284, identifier PROSPERO (CRD420261297284).

## 1 Introduction

Stroke is a major cause of death and disability worldwide. According to the Global Burden of Disease Study, stroke accounts for approximately 11% of all deaths and 5% of disability-adjusted life years worldwide (1). Despite advances in acute stroke care through treatments such as intravenous thrombolysis and endovascular thrombectomy, the proportion of patients with poor functional outcomes remains substantial. Emerging biomarkers may enhance the accuracy of outcome prediction and facilitate early, targeted interventions.

Neurofilament light chain (NfL) is an intermediate filament protein found primarily in large-diameter myelinated axons and is a major structural component of the neuronal cytoskeleton. It is essential for maintaining axonal integrity and function (2). Ischemic stroke induces energy failure and activates proteases, resulting in degradation of axonal neurofilaments, followed by the release of NfL into the extracellular space (3). Released NfL enters the cerebrospinal fluid and subsequently reaches peripheral blood; circulating concentrations therefore reflect the extent of axonal injury (3, 4). Furthermore, ischemia-induced changes in blood–brain barrier permeability facilitate the transfer of NfL from the central nervous system into the peripheral circulation (3, 5).

In ischemic stroke, several studies have investigated the association between blood NfL levels and functional outcomes, yet the findings remain inconsistent. For instance, some cohorts reported that higher blood NfL independently predicted poor 3-month functional outcomes (6, 7), whereas others found attenuated associations after adjustment for confounders or observed only modest predictive value (8, 9). These discrepancies may arise from differences in sample size, blood specimen type (serum vs. plasma), sampling timing, assay methods, and patient populations. This study aimed to systematically assess the association between blood NfL levels and functional outcomes in patients with ischemic stroke in an updated meta-analysis.

## 2 Methods

### 2.1 Study design

This systematic review and meta-analysis was conducted in accordance with the Preferred Reporting Items for Systematic Reviews and Meta-Analyses (PRISMA) statement (10). This study was performed in compliance with the Declaration of Helsinki. The requirement for informed consent and ethics approval was waived because no personal data were used. The study protocol was registered with International Prospective Register of Systematic Reviews (CRD420261297284).

### 2.2 Literature search

PubMed, Web of Science, Embase, Cochrane Library, and the China National Knowledge Infrastructure database were searched without language restrictions through March 15, 2026. Search terms were (“neurofilament light chain” OR “neurofilament-light chain” OR “NfL” OR “sNfL” OR “pNfL”) AND (“stroke” OR “ischemic stroke” OR “acute ischemic stroke” OR “cerebral stroke” OR “cerebrovascular accident” OR “brain vascular accident”). Additionally, we conducted a manual search of the reference lists of the identified articles and relevant reviews to identify additional studies that met the eligibility criteria.

### 2.3 Inclusion and exclusion criteria

Studies were eligible if: (1) they investigated the association between blood NfL and functional outcomes in patients with ischemic stroke, and (2) they provided sufficient data to calculate the odds ratios (ORs) and corresponding 95% confidence intervals (CIs).

The exclusion criteria comprised animal and *in vitro* experiments, conference abstracts, reviews, letters, case reports, systematic reviews, and meta-analyses; duplicate or overlapping cohorts from the same database; incomplete data; and unclear outcome measures.

### 2.4 Data extraction and quality assessment

Two reviewers independently screened the literature and extracted data. Extracted study characteristics included author, publication year, participant age, study location, study design, sample size, blood sample type, assay method, timing of blood collection, and timing of outcome assessment. Discrepancies were resolved by discussion or adjudication by a third reviewer.

Risk of bias and applicability concerns were assessed independently by two reviewers using the Quality Assessment of Diagnostic Accuracy Studies-2 (QUADAS-2) tool. QUADAS-2 assesses risk of bias across four domains: patient selection, index test, reference standard, and flow and timing. Judgments were categorized as “low”, “high”, or “unclear” risk. Disagreements were resolved by consensus.

### 2.5 Statistical analysis

Analyses were performed using Stata (version 18.0; StataCorp) software. Heterogeneity among studies was assessed using the *I*^2^ statistic and Cochran’s Q test. A random-effects model was applied when the heterogeneity test yielded *p* < 0.1 or *I*^2^ ≥ 50%; otherwise, a fixed-effects model was used. Subgroup analyses and meta-regression analysis were performed to explore sources of heterogeneity. Sensitivity analyses were performed to verify the robustness of the results. Publication bias was assessed using Begg’s and Egger’s tests, and the trim-and-fill method was employed if necessary. *p* ≤ 0.05 was considered significant.

## 3 Results

### 3.1 Literature search and selection

The study selection flow diagram is shown in Figure 1 and Supplementary Table S1. A total of 503 citations were identified. After removing 228 duplicate records, 275 records were screened by title and abstract, of which 247 were excluded as they did not meet the eligibility criteria. A total of 28 reports underwent full-text review. Subsequently, ten reports were excluded for the following reasons: animal study (*n* = 1), irrelevant topic (*n* = 2), unavailable data (*n* = 6), and duplicated data (*n* = 1) (11). Finally, 18 studies were included in the systematic review and meta-analysis (6–9, 12–25).

**Figure 1:**
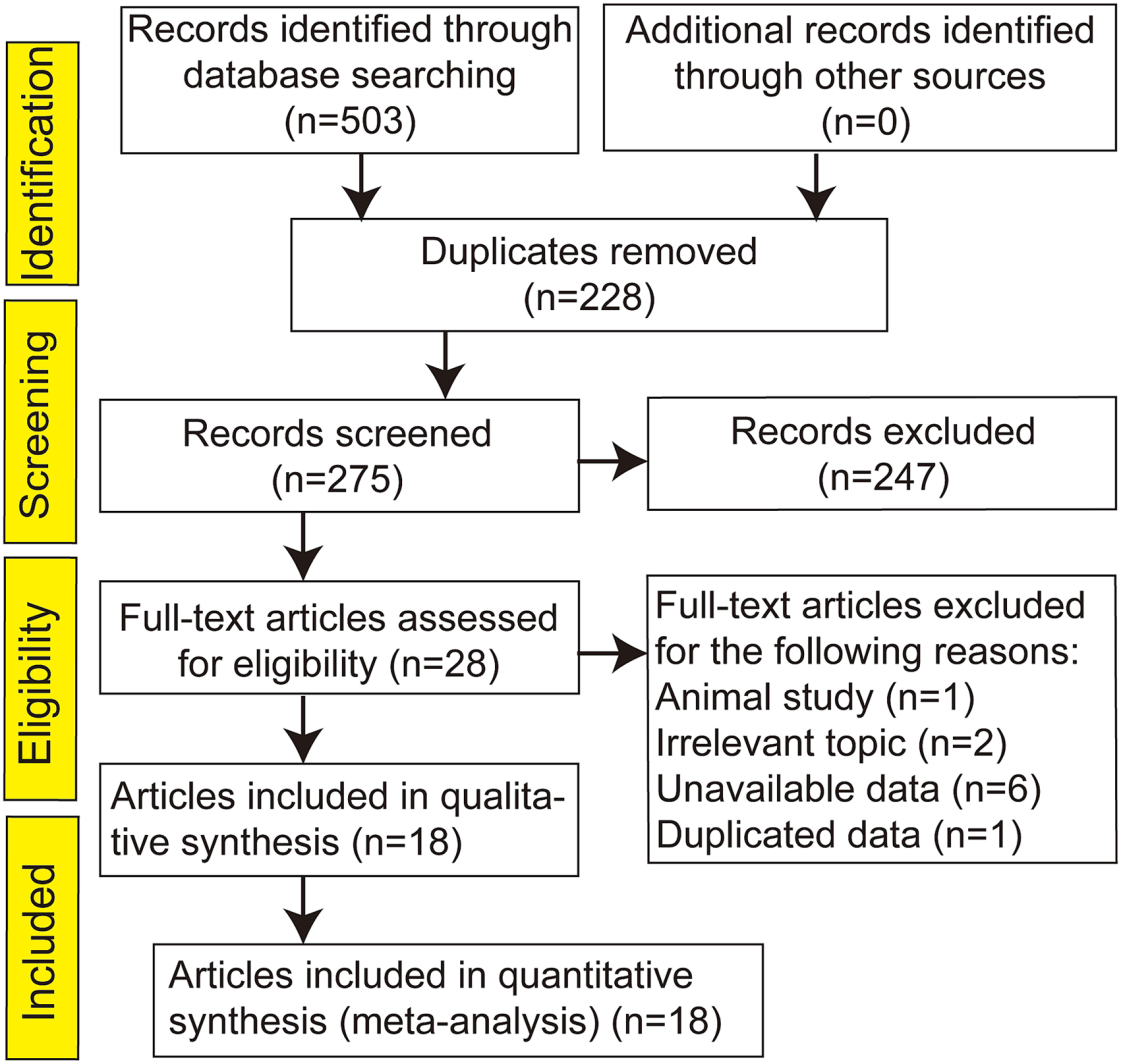
Flow diagram of the study selection process in the meta-analysis.

### 3.2 Characteristics of the included studies

The detailed characteristics of the included studies are listed in Table 1 (6–9, 12–25). These included studies were published between 2015 and 2025 (6–9, 12–25). 11 studies were conducted in Asia, six in Europe, and one in North America. Nine studies were prospective studies, and 9 studies were retrospective studies. Blood NfL was measured by single-molecule array (Simoa) in 8 studies, enzyme-linked immunosorbent assay (ELISA) in 8 studies, and electrochemiluminescence immunoassay (ECLIA) in the remaining 2 studies. Blood samples were collected at various time points, predominantly within 48 h after stroke onset, and functional outcomes were assessed at 3 months in most studies using the modified Rankin Scale (mRS), with poor outcomes generally defined as mRS 3–6. Serum was the most commonly used blood specimen (15 studies), followed by plasma (3 studies). Overall, 40% of patients exhibited unfavorable neurological outcomes.

**Table 1:** Characteristics of the included studies.

First author	Mean age (Year)	Male (%)	Sample size	Study design	Study location	NIHSS score	LAA occlusion (%)	Blood specimen	Assay method	Reperfusion therapy	Definition of poor functional outcome	Timepoint of blood specimen	Timepoint of outcome
Chen (13)	71.2	56.7	60	Pro	Taiwan	16.3	16.7	Plasma	SIMOA	100	mRS 3–6	Within 24 h	3 months
Chen (12)	65.7	60.2	93	Retro	China	NA	NA	Serum	ELISA	NA	NA	Within 36 h	3 months
De Marchis (8)	68^*^	63.7	504	Pro	Switzerland	5	16	Serum	ECLIA	37.7	mRS 3–6	Within 24 h	3 months
Duan (14)	63.3	52.3	153	Retro	China	NA	NA	Serum	ELISA	100	NA	Within 24 h	3 months
Gendron (15)	69.6	55.5	227	Retro	United States	6	20.7	Plasma	SIMOA	NA	mRS 4–6	Within 20 days	3 months
Guo (16)	63.0	59.3	113	Retro	China	NA	29.2	Serum	ELISA	NA	mRS 3–6	Within 24 h	3 months
Ji (17)	64.4	53.1	130	Retro	China	NA	NA	Serum	ELISA	NA	mRS 3–6	Within 48 h	3 months
Li (18)	67.7	68.4	152	Pro	China	2	32.2	Serum	SIMOA	19.7	NA	Within 24 h	3 months
Lin (19)	71^*^	63.6	110	Pro	China	16	36.4	Plasma	SIMOA	100	mRS 3–6	Within 24 h	3 months
Ma (20)	58.2	51.3	80	Retro	China	NA	NA	Serum	ELISA	NA	NIHSS ≥16	NA	3 months
Pedersen (21)	59^*^	64.0	595	Pro	Sweden	2.9	12.1	Serum	SIMOA	0.84	mRS 3–6	Within 14 days	3 months
Tiedt (22)	74.9	57.7	196	Pro	Germany	2	21.4	Serum	SIMOA	NA	NA	7 days	3 months
Traenka (9)	42^*^	59.2	49	Retro	Switzerland and Germany	NA	NA	Serum	ECLIA	NA	NA	Within 30 days	3 months
Uphaus (23)	68.7	58.8	211	Pro	Germany	3	15.2	Serum	SIMOA	NA	mRS 3–6	Within 24 h	3 months
Vollmuth (7)	76.1	53.1	213	Pro	Germany	13	12.2	Serum	ELISA	87.3	mRS 3–6	Within 24 h	3 months
Xu (24)	67.9	63.3	319	Pro	China	3.8	NA	Serum	ELISA	19.7	mRS 3–6	Within 3 days	3 months
Yang (6)	63.9^*^	68.4	133	Retro	China	NA	NA	Serum	ELISA	100	mRS 3–5	7 days	3 months
Zhou (25)	66^*^	59.3	110	Retro	China	8	NA	Serum	SIMOA	39.1	mRS 3–5	Within 7 days	6 months

*Indicates the median. ELISA, enzyme-linked immunosorbent assay; ECLIA, Electrochemiluminescence Immunoassay; LAA, large artery atherosclerosis; mRS, modified Rankin Scale; NA, not available; NIHSS, National Institutes of Health Stroke Scale; Pro, prospective; Retro, retrospective; SIMOA, single-molecule array.

### 3.3 Quality assessment

Supplementary Figures S1 and S2 present the results of the QUADAS-2 tool for assessing risk of bias and applicability concerns in individual studies. Patient selection was judged to have an unclear risk of bias in 13 studies primarily because they did not specify whether patients were enrolled consecutively. For the index-test domain, three studies had a high risk of bias and 15 had an unclear risk because blinding and the use of prespecified thresholds were not reported. For the reference-standard domain, 15 studies had an unclear risk because blinding was not reported. For flow and timing, five studies had a high risk because not all patients were included in the analysis. Applicability concerns were low for all studies.

### 3.4 Main results

ORs and corresponding 95% CIs were available from 16 studies and were included in the forest plot. The meta-analysis indicated significant heterogeneity (*I*^2^ = 87.5%, *p* < 0.01), so a random-effects model was used (Figure 2). The pooled estimate indicated that blood NfL was associated with poor functional outcomes after ischemic stroke (OR = 1.78, 95% CI: 1.41–2.24, *p* < 0.001).

**Figure 2:**
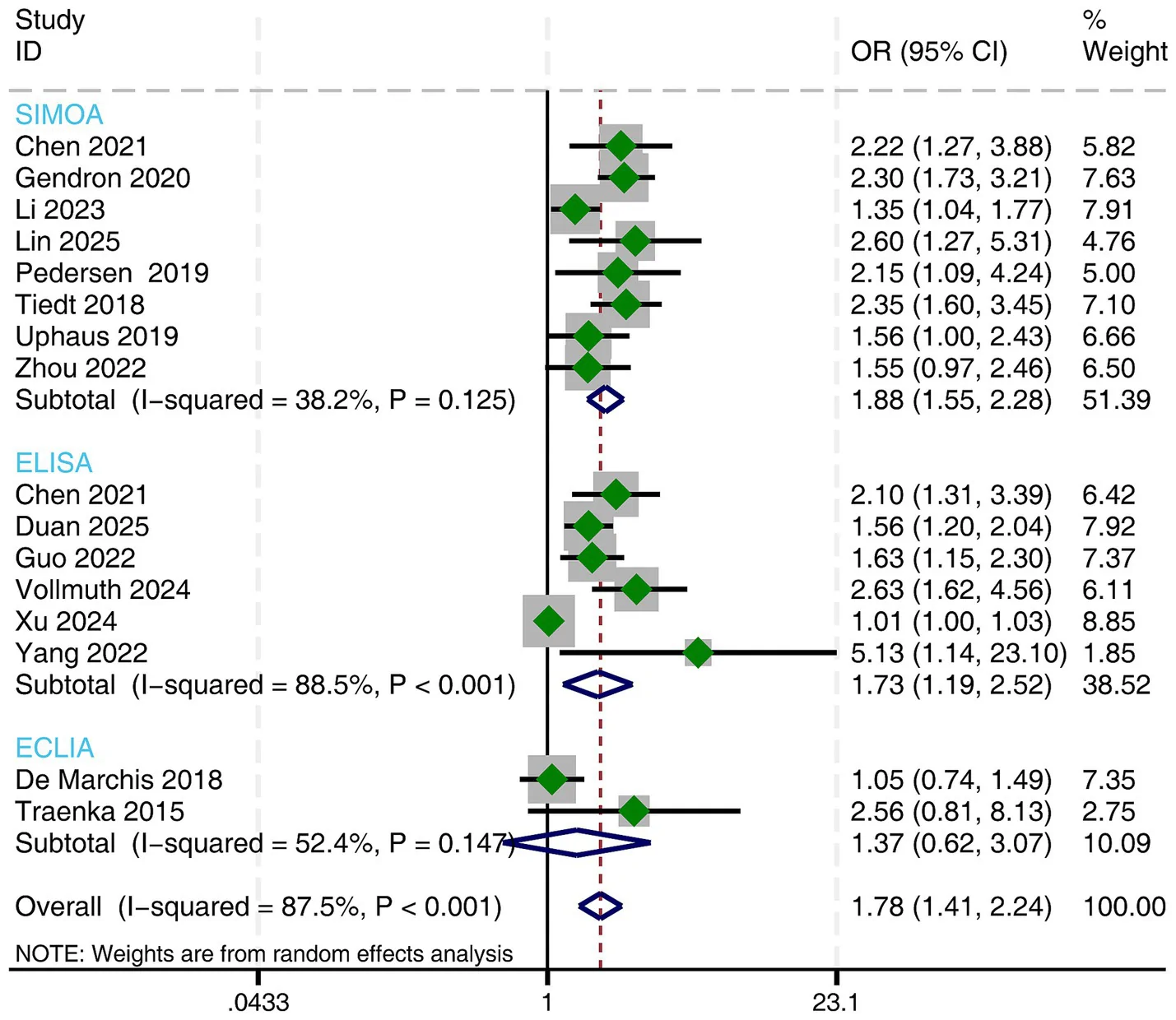
Forest plot of studies evaluating odds ratios for the association between elevated blood NfL and poor functional outcomes in patients with ischemic stroke. NfL, neurofilament light chain.

Sensitivity and specificity data from nine studies were included in the summary receiver operating characteristic analysis. The pooled area under the curve for blood NfL in predicting functional outcomes in patients with ischemic stroke was 0.79 (95% CI: 0.75–0.82), with 71% (95% CI: 62–79%) sensitivity and 74% (95% CI: 67–80%) specificity (Figure 3).

**Figure 3:**
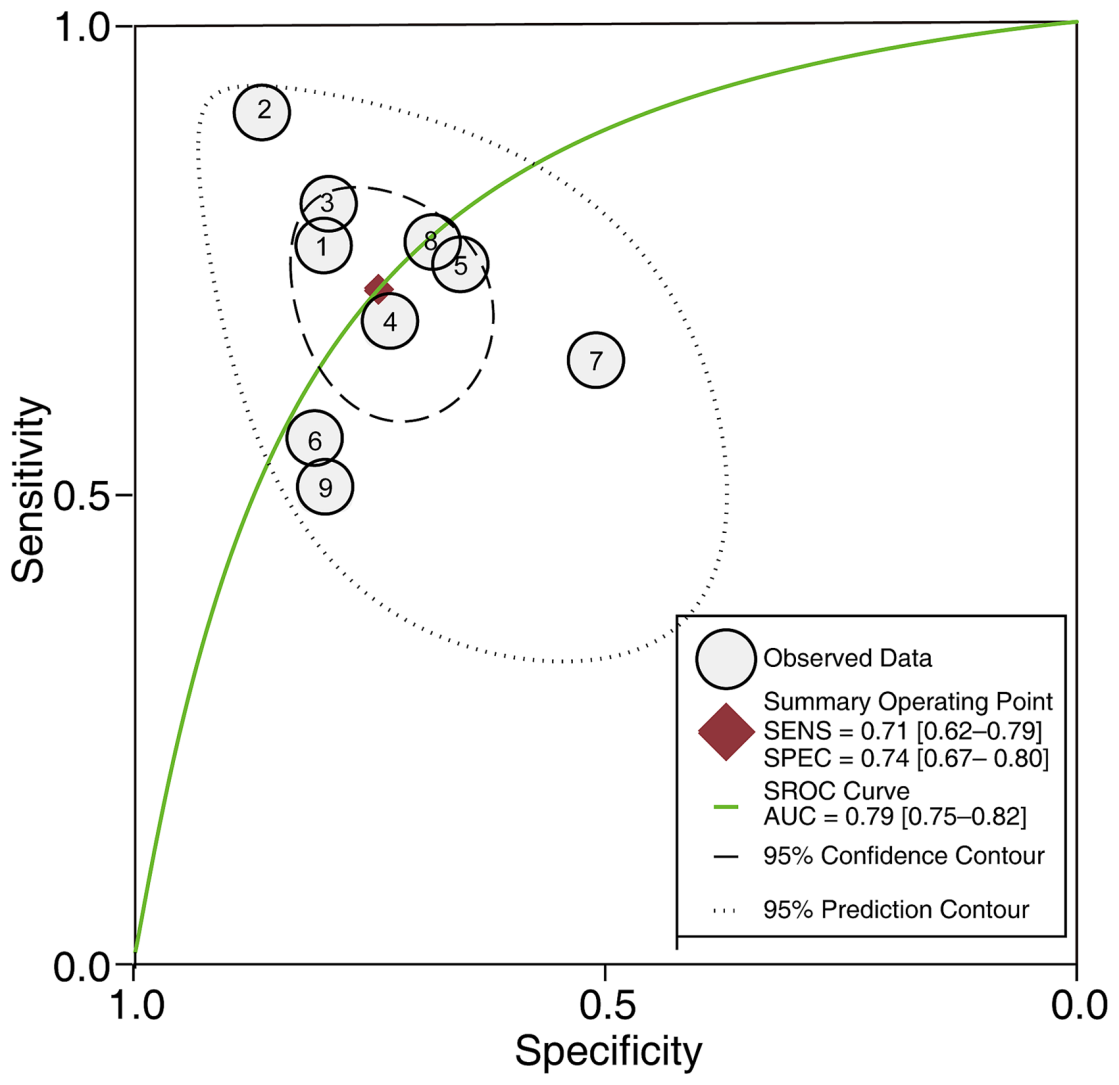
Summary receiver operating characteristic curve for blood NfL in predicting functional outcomes in patients with ischemic stroke. NfL, neurofilament light chain.

### 3.5 Subgroup analysis and meta-regression analysis

Subgroup analyses were conducted according to the mean age (≥66 years vs. <66 years), sample size (≥200 vs. <200), study design (prospective vs. retrospective), blood specimen type (serum vs. plasma), assay method (ELISA vs. Simoa vs. ECLIA), proportion of poor functional outcomes (≥40% vs. <40% vs. not available), blood sampling time (≥2 days vs. <2 days), reperfusion therapy rate (100% vs. <100% vs. not available), NIHSS score (≥6 vs. <6 vs. not available), definition of poor functional outcome (mRS 3–6 vs. others vs. not available), and proportion of large artery atherosclerosis (≥20% vs. <20% vs. not available). Neither the subgroup analyses (Supplementary Table S2) nor meta-regression analysis (Supplementary Table S3) identified a significant source of heterogeneity.

### 3.6 Sensitivity analysis and publication bias

Sensitivity analysis indicated that the pooled estimate was robust (Figure 4). Begg’s test did not indicate publication bias (*p* = 0.753; Figure 5A), whereas Egger’s test suggested potential publication bias (*p* < 0.001; Figure 5B). The trim-and-fill method did not materially alter the pooled estimate after four studies were imputed (adjusted OR = 1.61, 95% CI: 1.36–1.90; Supplementary Figure S3).

**Figure 4:**
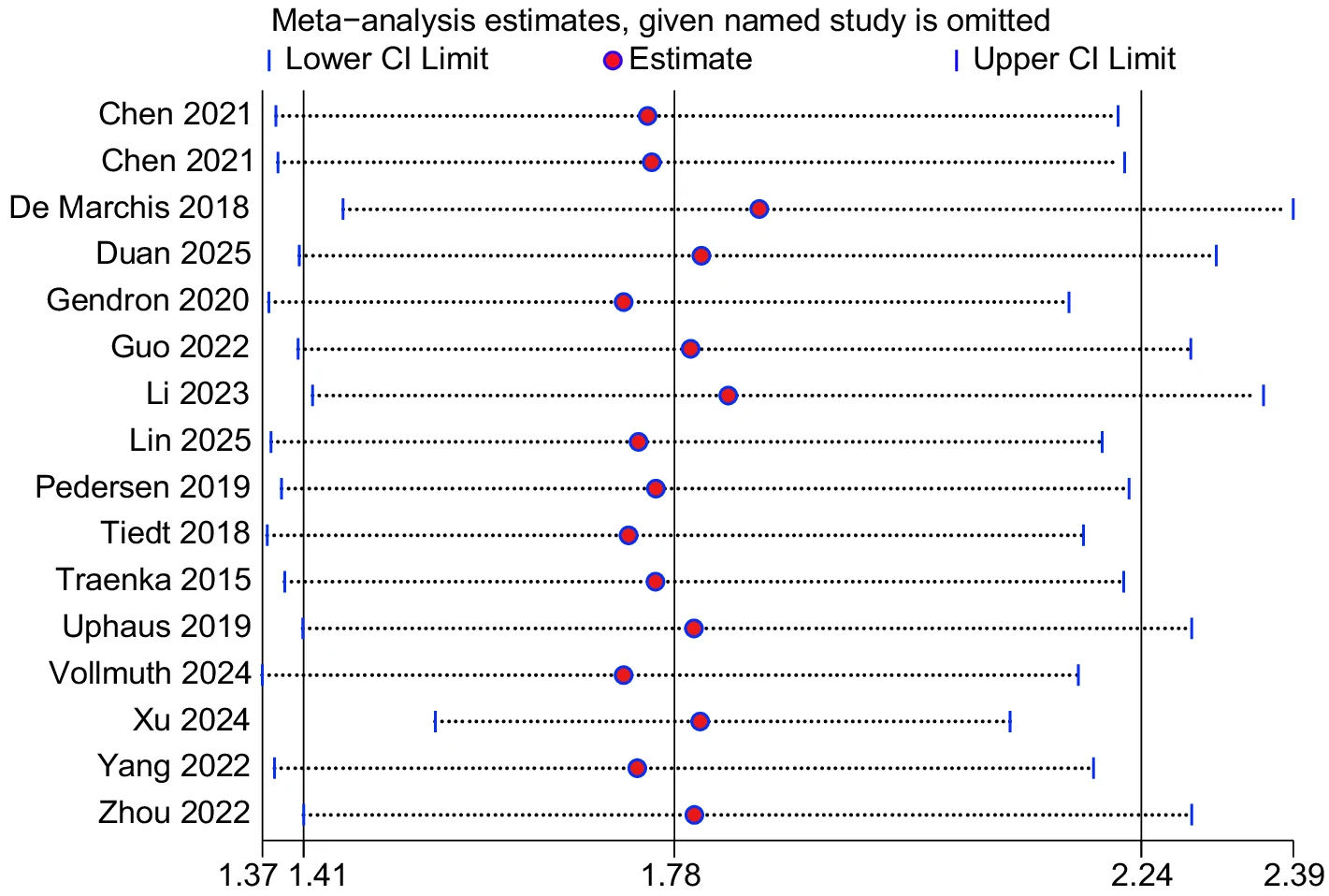
Sensitivity analysis of the association between elevated blood NfL and functional outcomes in patients with ischemic stroke. NfL, neurofilament light chain.

**Figure 5:**
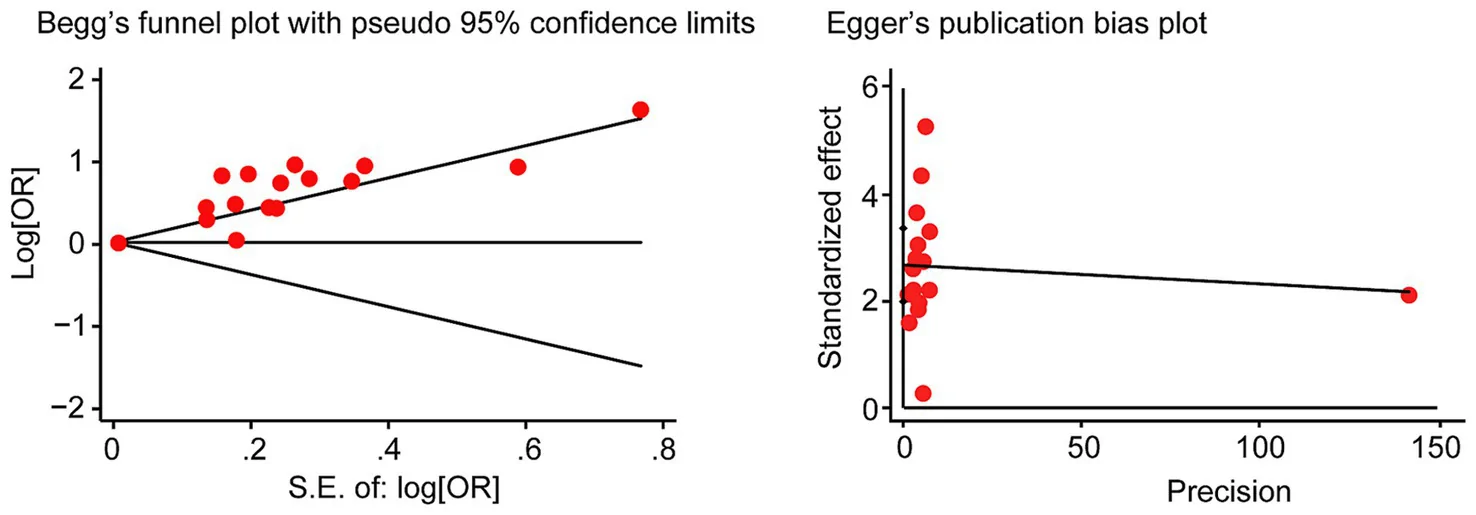
Tests for publication bias in the functional-outcome analysis.

## 4 Discussion

Stroke is a leading cause of long-term disability, and its prevalence is increasing. Studies have shown that the proportion of poor functional outcomes among patients with ischemic stroke ranges from one-third to one-half (26–28), consistent with the 40% observed across the included studies. The current meta-analysis identified an association between elevated blood NfL and an increased risk of poor functional outcomes (pooled OR = 1.78). To our knowledge, this is the meta-analysis to report that, for discriminating between good and poor functional outcomes, blood NfL yielded a pooled sensitivity of 71% and specificity of 74%.

A critical question for clinical translation is whether blood NfL provides prognostic information beyond established predictors—namely, NIHSS score, age, and infarct volume—and how it might be integrated into existing prognostic frameworks. A prospective cohort study by Vollmuth et al. (7) directly addressed this question in 213 patients with severe acute ischemic stroke. A baseline model comprising age and admission NIHSS score achieved an AUC of 0.68 for predicting poor 3-month functional outcome; adding serum NfL significantly improved the AUC to 0.84. When admission Alberta Stroke Program Early CT score and mechanical recanalization were included, the model had an AUC of 0.79. Adding serum NfL increased the AUC to 0.86, indicating that serum NfL may provide incremental prognostic value beyond a comprehensive set of clinical and imaging variables and may therefore be useful in multimarker prognostic models.

The utility of blood NfL as a biomarker depends partly on its temporal profile (29). Blood NfL levels progressively increase during the early subacute period (days 3–7) and may subsequently peak, suggesting that measurements obtained beyond 48 h after onset may better reflect the extent of secondary axonal degeneration. Our subgroup analysis showed that blood NfL was significantly associated with poor functional outcomes in both early (<2 days) and late (≥2 days) sampling subgroups, with a larger effect estimate in the latter (OR 1.65 vs. 1.94). This pattern suggests that sampling time may influence the observed association. Although NfL concentrations measured within 48 h were already associated with worse outcomes, measurements obtained after 2 days yielded a larger effect estimate, consistent with ongoing axonal release of NfL during the subacute phase (30). Notably, serial measurements can dynamically assess the trajectory of brain injury and may offer greater clinical value than a single measurement (21, 31).

The measurement of blood NfL commonly relies on three assay platforms: ELISA, ECLIA, and Simoa. ELISA is cost-effective and easy to operate, but its relatively low sensitivity makes it unsuitable for trace detection. Simoa has very high analytical sensitivity, extending to the femtomolar range; it is widely used in research and is increasingly being adopted in clinical settings. ECLIA generally offers greater analytical sensitivity than conventional ELISA and can provide rapid turnaround times at moderate cost (3, 32). Notably, our study found that ELISA and Simoa were significantly associated with functional outcomes, whereas the ECLIA subgroup did not show a statistically significant association. This finding may be attributable to the limited number of studies using ECLIA for blood NfL detection (only two included studies), and thus warrants confirmation in larger cohorts.

Although some individual-level findings from cohorts are encouraging, the interpretation of our pooled estimates is complicated by substantial between-study heterogeneity. Although meta-regression did not identify any single factor as a statistically significant moderator (all *p* > 0.05), several stratifications yielded marked reductions in within-group heterogeneity, pointing to possible sources of variation. Specifically, studies using the Simoa platform (*I*^2^ = 38.2%), those with smaller sample sizes (<200, *I*^2^ = 23.5%), retrospective designs (*I*^2^ = 12.6%), early blood sampling (<2 days, *I*^2^ = 46.7%), and higher stroke severity (NIHSS ≥6, *I*^2^ = 0.0%) all exhibited substantially lower heterogeneity compared with their counterparts. Nevertheless, substantial heterogeneity persisted in several other subgroups, indicating that the analyzed factors did not fully explain the overall heterogeneity. Other unmeasured confounders, such as disease severity, differences in study-center procedures, follow-up duration, and rates of loss to follow-up, may have contributed. The substantial overall heterogeneity limits the clinical interpretation of the pooled OR, as the prognostic value of blood NfL may be influenced by local laboratory protocols, case mix, and sampling timing. Therefore, current evidence supports a qualitative association rather than a precise quantitative threshold, and clinicians should interpret biomarker results in conjunction with established clinical scores rather than relying solely on blood NfL for outcome prediction.

This meta-analysis also had several limitations. First, 9 of the 18 studies used a retrospective design, which may introduce selection bias and residual confounding. Second, blood NfL is not pathologically specific and cannot distinguish neuronal damage caused by acute stroke from that attributable to preexisting neurologic comorbidities or other neurodegenerative processes (33). Third, some studies did not provide data on the proportion receiving reperfusion therapy or the proportion of poor functional outcomes, which limited the exploration of heterogeneity. Fourth, although risk of bias and applicability were assessed using QUADAS-2, this tool does not produce a quantitative summary score; therefore, the impact of overall methodological quality on the prognostic value of NfL remains unclear.

This meta-analysis found that elevated blood NfL levels are associated with poor functional outcomes in patients with ischemic stroke. Nevertheless, these findings should be interpreted with caution because of the substantial heterogeneity (*I*^2^ = 87.5%) and evidence of publication bias (Egger’s test, *p* < 0.001), which may have inflated the pooled effect estimate. Large-scale, well-designed prospective studies are needed to validate the independent prognostic value of blood NfL before clinical implementation.

## Data Availability

The original contributions presented in the study are included in the article/Supplementary material, further inquiries can be directed to the corresponding author.

## Supplementary material

SUPPLEMENTARY FIGURE S1 Risk of bias of the included studies.

SUPPLEMENTARY FIGURE S2 Risk of bias summary.

SUPPLEMENTARY FIGURE S3 Funnel plot after trim-and-fill adjustment for the functional-outcome analysis.

## References

[1] GBD 2021 Stroke Risk Factor Collaborators. Global, regional, and national burden of stroke and its risk factors, 1990–2021: a systematic analysis for the global burden of disease study 2021. Lancet Neurol. (2024) 23:973–1003. doi: 10.1016/s1474-4422(24)00369-7, 39304265 PMC12254192

[2] Gaetani L Blennow K Calabresi P Di Filippo M Parnetti L Zetterberg H. Neurofilament light chain as a biomarker in neurological disorders. J Neurol Neurosurg Psychiatry. (2019) 90:870–81. doi: 10.1136/jnnp-2018-320106, 30967444

[3] Liang Y Chen J Chen Y Wang Y Li L Xu Y . Advances on the neurofilament light chain as a biomarker in ischemic stroke. Neurol India. (2026) 74:S4–8. doi: 10.4103/neurol-india.Neurol-India-D-25-00124, 41633615

[4] Lanz TA Ruprecht K Somps CJ Göpfert JC Kam-Thong T Tekle F . Longitudinal evaluation of serum neurofilament light levels in normal healthy volunteers: defining a threshold of concern. J Neurol. (2025) 272:512. doi: 10.1007/s00415-025-13246-2, 40665000 PMC12263756

[5] Kalm M Boström M Sandelius Å Eriksson Y Ek CJ Blennow K . Serum concentrations of the axonal injury marker neurofilament light protein are not influenced by blood-brain barrier permeability. Brain Res. (2017) 1668:12–9. doi: 10.1016/j.brainres.2017.05.011, 28522263

[6] Yang YW Hu S Yang XY Sun Y Shi QY. Correlation between serum neurofilament light chain and prognosis of patients with acute ischemic stroke. J Hebei Med U. (2022) 24:271–6. doi: 10.19539/j.cnki.2095-2694.2022.04.004

[7] Vollmuth C Fiessler C Montellano FA Kollikowski AM Essig F Oeckl P . Incremental value of serum neurofilament light chain and glial fibrillary acidic protein as blood-based biomarkers for predicting functional outcome in severe acute ischemic stroke. Eur Stroke J. (2024) 9:751–62. doi: 10.1177/23969873241234436, 38400734 PMC11418447

[8] De Marchis GM Katan M Barro C Fladt J Traenka C Seiffge DJ . Serum neurofilament light chain in patients with acute cerebrovascular events. Eur J Neurol. (2018) 25:562–8. doi: 10.1111/ene.13554, 29281157

[9] Traenka C Disanto G Seiffge DJ Gensicke H Hert L Grond-Ginsbach C . Serum neurofilament light chain levels are associated with clinical characteristics and outcome in patients with cervical artery dissection. Cerebrovasc Dis. (2015) 40:222–7. doi: 10.1159/000440774, 26418549

[10] Page MJ McKenzie JE Bossuyt PM Boutron I Hoffmann TC Mulrow CD . The PRISMA 2020 statement: an updated guideline for reporting systematic reviews. Syst Rev. (2021) 10:89. doi: 10.1186/s13643-021-01626-4, 33781348 PMC8008539

[11] Barba L Vollmuth C Halbgebauer S Ungethüm K Hametner C Essig F . Prognostic serum biomarkers of synaptic, neuronal and glial injury in patients with acute ischemic stroke of the anterior circulation. Eur J Neurol. (2025) 32:e16581. doi: 10.1111/ene.16581, 39714176 PMC11664725

[12] Chen CH Cheng N Xiang CQ Peng W Li Y Zheng YQ. Expression of serum neurofilament light chain and ubiquitin carboxyl-terminal hydrolase-L1 in patients with acute ischemic stroke and their prognostic significance. Int J Lab Med. (2021) 42:2195–9. doi: 10.3969/j.issn.1673-4130.2021.18.005

[13] Chen CH Chu HJ Hwang YT Lin YH Lee CW Tang SC . Plasma neurofilament light chain level predicts outcomes in stroke patients receiving endovascular thrombectomy. J Neuroinflammation. (2021) 18:195. doi: 10.1186/s12974-021-02254-4, 34511123 PMC8436486

[14] Duan XF Zhang ZB Li H Lyu HY Qin JH Jia JD . Serum NfL and SIRT6 levels in patients with acute cerebral infarction and their relationship with disease severity and prognosis. Lab Med Clin. (2025) 22:2195–9. doi: 10.3969/j.issn.1672-9455.2025.16.012

[15] Gendron TF Badi MK Heckman MG Jansen-West KR Vilanilam GK Johnson PW . Plasma neurofilament light predicts mortality in patients with stroke. Sci Transl Med. (2020) 12:eaay1913. doi: 10.1126/scitranslmed.aay1913, 33177179 PMC9534269

[16] Guo GY Wang LY Wu R Shi Q Liu WH Hao T. The relationship between serum NFL and pNFH levels and the condition and prognosis of patients with acute ischemic stroke. Chin J Diffic and Compl Cas. (2022) 21:252–62. doi: 10.3969/j.issn.1671-6450.2022.03.007

[17] Ji K Wen J Zhong LJ Zeng DH. Changes of serum NfL,Galectin-3 and Netrin-1 levels in patients with acute ischemic stroke and their clinical significances. Labeled Immunoassays Clin Med. (2021) 28:1579–83. doi: 10.11748/bjmy.issn.1006-1703.2021.09.027

[18] Li J Zhang P Zhu Y Duan Y Liu S Fan J . Serum neurofilament light chain levels are associated with early neurological deterioration in minor ischemic stroke. Front Neurol. (2023) 14:1096358. doi: 10.3389/fneur.2023.1096358, 36970517 PMC10034185

[19] Lin K Wang Y Zhao W Zheng Y Sun X Fang L . Local cerebral neurofilament light chain during acute stage is associated with clinical outcomes in stroke patients receiving endovascular thrombectomy. BMC Neurol. (2025) 25:285. doi: 10.1186/s12883-025-04290-6, 40640701 PMC12243266

[20] Ma LN Mao LW Zhang AL. Correlation of serum IL-17A, TGF-β1, and NfL levels with neurological function in patients with ischemic stroke. Mod Diagn Treat. (2023) 34:1223–5.

[21] Pedersen A Stanne TM Nilsson S Klasson S Rosengren L Holmegaard L . Circulating neurofilament light in ischemic stroke: temporal profile and outcome prediction. J Neurol. (2019) 266:2796–806. doi: 10.1007/s00415-019-09477-9, 31375988 PMC6803587

[22] Tiedt S Duering M Barro C Kaya AG Boeck J Bode FJ . Serum neurofilament light: a biomarker of neuroaxonal injury after ischemic stroke. Neurology. (2018) 91:e1338–47. doi: 10.1212/wnl.0000000000006282, 30217937

[23] Uphaus T Bittner S Gröschel S Steffen F Muthuraman M Wasser K . NfL (neurofilament light chain) levels as a predictive marker for long-term outcome after ischemic stroke. Stroke. (2019) 50:3077–84. doi: 10.1161/strokeaha.119.026410, 31537188

[24] Xu C Yi T Qing T Jiang Y Yi X Xu J . Serum neurofilament light chain: a predictive marker for outcomes following mild-to-moderate ischemic stroke. Front Neurol. (2024) 15:1398826. doi: 10.3389/fneur.2024.1398826, 38841696 PMC11150679

[25] Zhou FY Chen DW Li HY Zhu C Shen YY Peng ZY . The association of serum neurofilament light chain and acute ischaemic stroke is influenced by effective revascularization. Dis Markers. (2022) 2022:5236080. doi: 10.1155/2022/5236080, 35557872 PMC9090527

[26] Bettger JP Thomas L Liang L Xian Y Bushnell CD Saver JL . Hospital variation in functional recovery after stroke. Circ Cardiovasc Qual Outcomes. (2017) 10:e002391. doi: 10.1161/circoutcomes.115.002391, 28096203

[27] Dong A Maier B Guillon B Preterre C De Gaalon S Gory B . TICI-RANKIN mismatch: poor clinical outcome despite complete endovascular reperfusion in the ETIS registry. Rev Neurol (Paris). (2023) 179:230–7. doi: 10.1016/j.neurol.2022.10.003, 36804012

[28] Park TH Park SS Ko Y Lee SJ Lee KB Lee J . The iScore predicts clinical response to tissue plasminogen activator in Korean stroke patients. J Stroke Cerebrovasc Dis. (2014) 23:367–73. doi: 10.1016/j.jstrokecerebrovasdis.2013.05.025, 23800501

[29] Sanchez JD Martirosian RA Mun KT Chong DS Llorente IL Uphaus T . Temporal patterning of Neurofilament light as a blood-based biomarker for stroke: a systematic review and Meta-analysis. Front Neurol. (2022) 13:841898. doi: 10.3389/fneur.2022.841898, 35651349 PMC9149427

[30] Mathias K Tiscoski ADB Vuolo F Cechinel MA Andrade NM Petronilho F. From bench to bedside: neurofilament light chain as a biomarker in ischemic stroke. Curr Med Sci. (2026) 46:604–15. doi: 10.1007/s11596-026-00200-0, 42047916 PMC13314808

[31] Correia M Silva I Gabriel D Simrén J Carneiro A Ribeiro S . Early plasma biomarker dynamic profiles are associated with acute ischemic stroke outcomes. Eur J Neurol. (2022) 29:1630–42. doi: 10.1111/ene.15273, 35124870

[32] Vecchio D Puricelli C Malucchi S Virgilio E Martire S Perga S . Serum and cerebrospinal fluid neurofilament light chains measured by SIMOA™, Ella™, and Lumipulse™ in multiple sclerosis naïve patients. Mult Scler Relat Disord. (2024) 82:105412. doi: 10.1016/j.msard.2023.105412, 38198989

[33] Narayanan S Shanker A Khera T Subramaniam B. Neurofilament light: a narrative review on biomarker utility. Fac Rev. (2021) 10:46. doi: 10.12703/r/10-46, 34131656 PMC8170685

