# Supplementary material for: Prognostic performance of blood neurofilament light chain for functional outcomes in patients with ischemic stroke: a systematic review and meta-analysis
Source: Front Neurol. 2026 Oct 12;17:1895703. doi: 10.3389/fneur.2026.1895703 (PMC13696241; doi:10.3389/fneur.2026.1895703)
Supplement: Supplementary file 1 [file Table_1.doc]

**Table S1:** Electronic database search strategies and study selection

| **Search strategies** | |
| --- | --- |
| Search date | 15th March 2026 |
| Search date range | From inception to 15th March 2026 |
| Language restrictions | None |
| Study types | None |
| **PubMed (https://pubmed.ncbi.nlm.nih.gov/)** | |
| Search results | 141 |
| Search terms | (“neurofilament light chain[Title]” OR “neurofilament-light[Title/Abstract]” OR “NfL[Abstract]” OR “sNfL[Abstract]” OR “pNfL[Abstract]”) AND (“stroke[Title]” OR “ischemic stroke[Title]” OR “acute ischemic stroke[Title]” OR “cerebral stroke[Title/Abstract]” OR “cerebrovascular accident[Title/Abstract]” OR “brain vascular accident[Title/Abstract]”) |
| **Embase (https://www.embase.com/)** | |
| Search results | 232 |
| Search terms | ("neurofilament light chain".ti,ab. OR "neurofilament-light".ti,ab. OR NfL.ti,ab. OR sNfL.ti,ab. OR pNfL.ti,ab.) AND ("stroke".ti,ab. OR "ischemic stroke".ti,ab. OR "acute ischemic stroke".ti,ab. OR "cerebral stroke".ti,ab. OR "cerebrovascular accident".ti,ab. OR "brain vascular accident".ti,ab.) |
| **Web of science (http://apps.webofknowledge.com)** | |
| Search results | 77 |
| Search terms | (TI=("neurofilament light chain" OR "neurofilament-light" OR NfL OR sNfL OR pNfL) OR AB=("neurofilament light chain" OR "neurofilament-light" OR NfL OR sNfL OR pNfL)) AND (TI=("stroke" OR "ischemic stroke" OR "acute ischemic stroke" OR "cerebral stroke" OR "cerebrovascular accident" OR "brain vascular accident") OR AB=("stroke" OR "ischemic stroke" OR "acute ischemic stroke" OR "cerebral stroke" OR "cerebrovascular accident" OR "brain vascular accident")) |
| **Cochrane Library (https://www.cochranelibrary.com)** | |
| Search results | 13 |
| Search terms | (("neurofilament light chain" OR "neurofilament-light" OR NfL OR sNfL OR pNfL):ti,ab) AND (("stroke" OR "ischemic stroke" OR "acute ischemic stroke" OR "cerebral stroke" OR "cerebrovascular accident" OR "brain vascular accident"):ti,ab) |
| **China national knowledge infrastructure (CNKI) (http://www.chkd.cnki.net)** | |
| Search results | 40 |
| Search terms | (Title:'neurofilament light chain' OR Title:'neurofilament protein light chain' OR Abstract:'NfL' OR Abstract:'sNfL' OR Abstract:'pNfL') AND (Title:'stroke' OR Title:'ischemic stroke' OR Title:'ischemic cerebral stroke' OR Abstract:'cerebral infarction' OR Abstract:'cerebral stroke' OR Abstract:'cerebrovascular accident') |
