# Supplementary material for: Prognostic performance of blood neurofilament light chain for functional outcomes in patients with ischemic stroke: a systematic review and meta-analysis
Source: Front Neurol. 2026 Oct 12;17:1895703. doi: 10.3389/fneur.2026.1895703 (PMC13696241; doi:10.3389/fneur.2026.1895703)
Supplement: Supplementary file 2 [file Table_2.doc]

**Table S2** Subgroup analysis of the association between NfL and functional outcomes in patients with ischemic stroke

|  | N | OR | 95% CI | *p* value | *I*^2^ (*p* value) |
| --- | --- | --- | --- | --- | --- |
| Participant age (years) |  |  |  |  |  |
| ≥66 | 10 | 1.70 | 1.28–2.26 | <0.001 | 89.2 (<0.001) |
| <66 | 6 | 1.74 | 1.45–2.08 | <0.001 | 0.00 (0.524) |
| Sample size |  |  |  |  |  |
| ≥200 | 6 | 1.61 | 1.09–2.37 | 0.016 | 89.6 (<0.001) |
| <200 | 10 | 1.77 | 1.51–2.07 | <0.001 | 23.5 (0.227) |
| Study design |  |  |  |  |  |
| Prospective study | 9 | 1.67 | 1.25–2.23 | 0.001 | 86.2 (<0.001) |
| Retrospective study | 7 | 1.83 | 1.54–2.17 | <0.001 | 12.6 (0.334) |
| Blood specimen |  |  |  |  |  |
| Serum | 13 | 1.65 | 1.31–2.08 | <0.001 | 85.0 (<0.001) |
| Plasma | 3 | 2.32 | 1.89–2.99 | <0.001 | 0.00 (0.941) |
| Assay method |  |  |  |  |  |
| ELISA | 6 | 1.73 | 1.19–2.52 | 0.004 | 88.5 (<0.001) |
| Simoa | 8 | 1.88 | 1.55–2.28 | <0.001 | 38.2 (0.125) |
| ECLIA | 2 | 1.37 | 0.62–3.07 | 0.437 | 52.4 (0.147) |
| Proportion of poor functional outcomes (%) |  |  |  |  |  |
| ≥40% | 4 | 2.34 | 1.78–3.08 | <0.001 | 0.0 (0.918) |
| <40% | 8 | 1.48 | 1.13–1.92 | 0.004 | 87.6 (<0.001) |
| NA | 4 | 2.04 | 1.56–2.65 | <0.001 | 0.0 (0.564) |
| Timepoint of blood specimen (days) |  |  |  |  |  |
| ≥2 | 7 | 1.94 | 1.23–3.07 | 0.005 | 90.0 (<0.001) |
| <2 | 9 | 1.65 | 1.37–1.98 | <0.001 | 46.7 (0.059) |
| Reperfusion therapy rate (%) |  |  |  |  |  |
| 100% | 4 | 1.99 | 1.40–2.82 | <0.001 | 33.3 (0.212) |
| <100% | 6 | 1.40 | 1.07–1.85 | 0.016 | 80.2 (<0.001) |
| NA | 6 | 2.00 | 1.69–2.36 | <0.001 | 0.0 (0.515) |
| NIHSS score |  |  |  |  |  |
| ≥6 | 5 | 2.19 | 1.78–2.68 | <0.001 | 0.0 (0.570) |
| <6 | 6 | 1.42 | 1.07–1.89 | 0.017 | 83.9 (<0.001) |
| NA | 5 | 1.71 | 1.42–2.06 | <0.001 | 0.0 (0.437) |
| Definition of poor functional outcome |  |  |  |  |  |
| mRS 3–6 | 7 | 1.66 | 1.18–2.34 | 0.004 | 84.5 (<0.001) |
| Other | 4 | 1.91 | 1.42–2.58 | <0.001 | 34.7 (0.204) |
| NA | 5 | 1.74 | 1.38–2.19 | <0.001 | 44.1 (0.128) |
| Proportion of LAA |  |  |  |  |  |
| ≥20% | 5 | 1.89 | 1.46–2.45 | <0.001 | 60.4 (0.039) |
| <20% | 5 | 1.76 | 1.22–2.54 | 0.003 | 64.2 (0.025) |
| NA | 6 | 1.61 | 1.12–2.32 | 0.010 | 82.9 (<0.001) |

CI, confidence interval; ECLIA, electrochemiluminescence immunoassay; ELISA, enzyme-linked immunosorbent assay; LAA, large artery atherosclerosis; mRS, modified rankin scale; N, number; NA, not available; NIHSS, national institutes of health stroke scale; OR, odds ratio; Simoa, single-molecule array.
