# Supplementary material for: Prognostic performance of blood neurofilament light chain for functional outcomes in patients with ischemic stroke: a systematic review and meta-analysis
Source: Front Neurol. 2026 Oct 12;17:1895703. doi: 10.3389/fneur.2026.1895703 (PMC13696241; doi:10.3389/fneur.2026.1895703)
Supplement: Supplementary file 3 [file Table_3.doc]

**Table S3** Meta-regression analysis of NfL predicting functional outcomes in patients with ischemic stroke

| Heterogeneity factors | Coefficient | SE | *t* | *p* value | 95% CI |
| --- | --- | --- | --- | --- | --- |
| Participant age | 0.332 | 0.326 | 1.02 | 0.366 | -0.573–1.238 |
| Sample size | 0.254 | 0.204 | 1.25 | 0.280 | -0.311–0.819 |
| Study design | 0.509 | 0.295 | 1.73 | 0.159 | -0.309–1.327 |
| Blood specimen | -0.193 | 0.242 | -0.80 | 0.470 | -0.864–0.478 |
| Assay method | -0.109 | 0.085 | -1.29 | 0.267 | -0.344–0.126 |
| Proportion of poor functional outcomes | 0.124 | 0.078 | 1.58 | 0.190 | -0.094–0.341 |
| Timepoint of blood specimen | 0.334 | 0.315 | 1.06 | 0.349 | -0.541–1.209 |
| Reperfusion therapy rate | -0.026 | 0.063 | -0.42 | 0.697 | -0.200–0.148 |
| NIHSS score | 0.537 | 0.230 | 2.33 | 0.080 | -0.103–1.177 |
| Definition of poor functional outcome | 0.174 | 0.091 | 1.90 | 0.130 | -0.080–0.427 |
| Proportion of LAA | -0.193 | 0.101 | -1.92 | 0.127 | -0.473–0.086 |

NIHSS, National Institutes of Health Stroke Scale; NfL, neurofilament light chain; LAA, large artery atherosclerosis; SE, standard error; CI, confidence interval.
